# Does Scale and Efficiency of Government Health Expenditure Promote Development of the Health Industry?

**DOI:** 10.3390/ijerph17155529

**Published:** 2020-07-30

**Authors:** Mengying Wang, Stuart Gilmour, Chunhai Tao, Kaixuan Zhuang

**Affiliations:** 1 School of Statistics, Jiangxi University of Finance and Economics, No.169, East Shuanggang Road, Changbei, Nanchang 330013, China; 2201810078@stu.jxufe.edu.cn (M.W.); 2201921840@stu.jxufe.edu.cn (K.Z.); 2 Graduate School of Public Health, St. Luke’s International University, 5th Floor, Omura Susumu and Mieko Memorial Chuo-ku, Tokyo 105-0045, Japan

**Keywords:** macro-economic development, health industry, government health expenditure, efficiency, semi parametric generalized additive model

## Abstract

Macro-economic development of China’s health industry is essential to the sustainable development and growth momentum of the national economy. Strategies to promote the development and rebalancing of the industrial structure need to be improved in order to transform China’s health industry and drive development. Based on panel data of 25 regions in China from 2004 to 2016, this paper analyzes the linear and non-linear relationship between Chinese government health expenditure (GHE), GHE efficiency, and the macro-economic development of the health industry. It uses a novel index of industrial structure to measure the transformation of industrial sectors in China, based on a semi-parametric generalized additive model. The model shows that per capita GHE and its efficiency have a significant positive linear and comprehensive non-linear effect on the development of health industry structure. By analyzing the interaction of GHE and its efficiency, we show that high expenditure with low-efficiency regimes and high expenditure with high-efficiency regimes have a positive impact on the development of industrial structure. Following the empirical results, the paper puts forward corresponding policy suggestions for the role of fiscal policy in promoting the development of the health industry in China.

## 1. Introduction

Health is the necessary requirement to improve people’s quality of life and promote the development of the economy and society. The 18th National Congress of the Communist Party of China promulgated the Healthy China 2030 plan (hereinafter referred to as the Outline) [1] and established the “big health concept” with “health promotion as the center”. The Outline proposed that China should establish a comprehensive health industry and optimize its structure, and the health industry should become a pillar of the national economy, meet the public’s demand for health and improve the quality of health care. At the same time, as an emerging industry, it should contribute to expanding domestic demand and stimulating economic development. In 2015, the global market size of the health industry reached 7.98 trillion dollars, accounting for 10.3% of global GDP. The United States accounts for 2.98% of global health spending, the European Union accounts 1.8%, and China only 0.79% despite its large population. The distribution of major health industries in China is also quite different to other developed countries. More than 50% of China’s health industry is the drug market, while this proportion in the United States is less than 15% [2]. Throughout the development of China’s health industry, structural deviation among the three sectors of industry, and imbalances in the internal structure of the industry, have presented challenging problems. Therefore, optimization of the health industrial structure is of great significance for national health promotion and the smooth realization of economic transformation. Understanding this industrial structure and how the distribution of sectors within the industry affects the development of China’s health industry is essential to realizing the goals laid out in the Outline, and to improving the health of the Chinese population.

The health industry originated from the concept of health management, initially formed in the 1960s in the United States, followed by Japan and Europe. With the establishment of the first batch of health management companies in 2000, China formally developed a modern health industry. Health management refers to a process of overall management of health risk factors of individuals or people. Its purpose is to mobilize the enthusiasm of individuals and groups, and effectively use limited resources to achieve the maximum health effect. The Outline emphasizes that the development of China’s health industry should be changed from the narrow definition of “medical and health service industry” to the broad definition of “comprehensive health industry”. This definition includes not only the medical and health service industries, but also industries aimed at disease prevention, treatment, maintenance and recovery.

Following the typical classification of national economies, China’s health industry can be divided into three sectors. The three-sector model in economics divides economies into three sectors of activity: extraction of raw materials (primary), manufacturing (secondary), and services (tertiary). According to the model, the main focus of an economy’ s activity shifts from the primary, through the secondary and finally to the tertiary sector. The primary sector covers organic agriculture, Chinese herbal medicine cultivation and other activities; the secondary sector covers pharmaceutical manufacturing, health foods, health equipment and equipment manufacturing; while the tertiary sector covers health services, health management, health financing, environmental and public facilities, and other ancillary management functions. In describing health industry development, we focus on the evolution of industrial structure. With economic and technological progress, the resource utilization level constantly breaks through past boundaries, so as to continuously promote the growth of sunrise industries. In the health industry, Chinese medicinal agriculture is gradually being supplanted by more technologically advanced health sectors, such as pharmaceutical manufacturing and health care services. In researching this development, we seek tools to quantify the shift from primary production through secondary production to tertiary industry and services, within the specific characteristics of the healthcare context. It can be seen that the development of the health industry will lead to a change in its industrial structure that will in turn accelerate the promotion of health equity and accessibility, and achieve a higher level of health [3]. Development of the industry is partly an endogenous process in which the market optimizes resource allocation leading to the development of industrial structure, but fiscal policy and its efficient application by the government also play an important role [4,5].

## 2. Literature Review

Current research on the impact of fiscal policy on the development of industrial structure can be divided into two general areas. The first is research on the influence of fiscal expenditure scale on the development of industrial structure, which has not reached a unanimous conclusion. Some scholars think that the magnitude of fiscal expenditure has a positive impact on the development of industrial structure [6,7], while others think that the fiscal expenditure scale has a negative impact on the development of industrial structure [8,9]. Most of the models used in these studies are econometric models that rely on constant parameters. There are also a small number of scholars who have explored the impact of fiscal expenditure scale on the development of industrial structure from a non-linear perspective [10]. They have discussed the impact of fiscal expenditure scale on the development of industrial structure from multiple perspectives such as economic zone systems [11,12] and spatial effects [13,14]. The second area of research on industrial development explores the influence of the structure of fiscal expenditure on industrial development. Scholars generally think that the fiscal expenditure structure plays an important role in promoting China’s industries [15], and some conclude that public expenditure will promote the development of industrial structure [16,17]. As a component of financial expenditure, public expenditure is an external self-regulating force with the government as the main regulating body, whether through direct financial investment or indirect financial subsidies and government purchases. Yan (2016) [18] pointed out that financial expenditure affects the change of industrial structure through public expenditure and both agricultural and non-agricultural productive expenditure. In general, to date research on the influence of fiscal expenditure scale and fiscal expenditure structure on the development of industrial structure ignores the influence of efficiency of fiscal expenditure. By investigating only the effect of scale and structure of financial expenditure we cannot understand the influence of internal mechanisms of financial expenditure on the development process.

As a part of public expenditure in financial expenditure, government health expenditure reduces personal medical expenses through transfer payments, and reduces the price of health services, which improves health outcomes and contributes to industrial development [19]. Therefore, it is important to study the impact of government health expenditure on the development of health industrial structure. Previously, Fedotenkov and Gupta (2020) [20] found non-significant effects of health expenditure on industry and services, but their paper studied this phenomenon at the national level where significant provincial differences can obscure large effects through confounding. In this paper, we expand on their work by considering the role of these factors at the provincial level. In addition, when we study the impact of government health expenditure, if we only consider its scale and do not consider expenditure efficiency there is likely to be bias in the measurement results.

## 3. Objectives

The existing literature on the impact of fiscal expenditure on industrial structure development has not reached a consensus. Most of the models used in the research rely on constant parameter econometric models, or ignore the impact of fiscal expenditure efficiency. This paper aims to analyze the effect of government health expenditure and the efficiency of that expenditure on development of the health industry. Using data from 25 provinces and cities in China from 2004 to 2016, we analyze the relationship between per capita government health expenditure (GHE), GHE efficiency and the development of the structure of the health industry. This paper uses a variable parameter econometric model to construct and optimize an index of industrial structure development.

This paper adds to current knowledge about health industrial development by considering both the impact of per capita government health expenditure and government health expenditure efficiency on the development of health industrial structure. The empirical analysis uses a semi parametric generalized additive model, which differs from the single linear or non-linear relationships used to date. By combining linear and non-linear measurement models, it is possible to provide a theoretical basis and practical guidance for the development of health industrial structure.

The structure of this paper is as follows. Part 4 describes the data and variables used in this paper. In part 5, we use the semi parametric additive model to measure the non-linear impact of per capita government health expenditure and government health expenditure efficiency on the development of health industrial structure. Part 6 discusses the development of health industry based on the empirical results of part 5 and makes policy recommendations to optimize the development of China’s health industrial structure.

## 4. Data and Model Description

### 4.1. Data Description

This paper uses a development index (DI) as the response variable when measuring structural composition of the health industry, and local per capita government health expenditure and government health expenditure efficiency as the main explanatory variables. At the same time, on the basis of reference to the existing research on factors influencing industrial structure development, we incorporate variables that measure technological progress, human capital, population distribution, per capita private health expenditure, aging and time as control variables.

#### 4.1.1. Response Variable

Industrial development is a complex dynamic process. The increase in the overall scale and the proportion of the tertiary sector do not represent the development of the industry, and excessive increase in the output value and proportion of the tertiary sector will lead to unbalanced development. Therefore, attention needs to be paid to coordination among the three sectirs of industry, in order to promote the sustainable development of China’s economy through the mutual promotion and coordinated development of these three sectors.

There are different methods to measure the development index of industrial structure in the existing research, such as using the ratio of the output value of the tertiary sector to the output value of the secondary sector [21], or using the proportion of the financial industry within the tertiary sector [22]. The methods used in [21,22] ignore the overall impact of structural changes in the secondary and tertiary sectors. The index used in this paper measures the process of changing the focus of health industrial structure from the primary to the secondary and tertiary sectors with the development of the economy. It can be noted that the larger the DI, the higher the level of industrial structure development.

We use a measure of industrial development from John H. Moore [23], which calculates the proportion of the economy in the three sectors and then transforms it into a type of measure in a radial space. See Appendix A for more details of the development of this index, which we hereafter refer to as the DI.

#### 4.1.2. Main Explanatory Variables

This study aims to understand the effect of government health expenditure and efficiency of government health expenditure on industrial development. We incorporate local per capita government health expenditure scale (PGHE) as the first explanatory variable. This is measured by local government health expenditure divided by the total population of the region. Health expenditure is measured using the super efficiency slacked based model under variable returns to scale (Super-SBM-VRS Model). We use the rate of moderate and severe malnutrition and child mortality as a proxy for the health level of residents, and the number of outpatient clinics and residents with access to medical institutions as a measure of health system capacity. The hospital’s per capita medical expenses, which reflect the incidence and mortality of class A and class B legally reported infectious diseases provided by the government’s basic public medical services, and the utilization rate of beds, which reflects the quality of medical services, are output indicators to measure the efficiency of government health expenditure [24]. All explanatory variables and the development of the Super-SBM-VRS model are presented in Appendix A.

#### 4.1.3. Control Variables

Because the effect of government expenditure is confounded by many other social and economic phenomena, we include several control variables in our models. These are listed below

- Technological progress (TP): the development of the industry is closely related to the degree of technological progress. Therefore, this paper uses the ratio of the number of patents to the number of pharmaceutical manufacturing enterprises to reflect the level of technological progress. This variable primarily measures technical progress in the secondary sector of the health industry, but still has utility in measuring the role of innovation in enhancing industrial development, since the DI used in this study incorporates changes in all three sectors simultaneously.
- Population skewedness (PS): with industrialization and urbanization in China there has been significant movement of people into cities from rural provinces, with associated impacts on urban growth, inequality, and health outcomes. Considering that the permanent population is smaller than the registered population, this will lead to labor migration, and population aggregation will expand the regional economic gap and promote cross regional agglomeration. We measure the degree of population deviation as the ratio of the permanent population to the population of registered households.
- Human capital (HC): The variable reflecting level of human capital in this paper is the average years of education in each region.
- Economic gap (EG): We use the ratio of per capita GDP in each region to per capita GDP of the whole country to measure the economic gap at the regional level.
- Per capita private health expenditure (PPHE): the expenditure of residents in health care has an important impact on the development of health industrial structure. This paper selects per capita private health expenditure to measure the consumption of residents’ medical services.
- Old-age Dependency Ratio (ODR): Aging is occurring rapidly in some parts of China and also reflects the depopulation of some areas due to urbanization. We measure this using the old-age population dependency ratio, which is the ratio of people aged over 65 years old to the population aged 15-64 years old.
- Year: We also allow the change in the index of industrial structure to change with time, measured as years. Data of 13 years from 2004 to 2016 are selected.

#### 4.1.4. Data Sources

In view of the lack of data in Beijing, Tianjin, Shanghai, Hainan, Qinghai and Tibet, this paper selects 25 other regions in China from 2004 to 2016 as data samples. The original data of all indicators are from the China statistical yearbook of tertiary industry [25], the Almanac of China’s Finance and Banking [26], China statistical yearbook [27] and the China health statistical yearbook [28]. The descriptive statistical analysis results of each variable are shown in Table 1.

### 4.2. Model Introduction

#### Super Efficiency Slacked Based Model (SBM) under the Variable Returns to Scale (Super-SBM-VRS) Model

Data Envelopment Analysis (DEA) is the first non-parametric efficiency evaluation method proposed by Charnes and Cooper (1978) [29]. In 1993, Andersen [30] constructed the super efficiency DEA model, which resolved some problems in the original model and made the efficiency of decision units comparable. In practice, considering that some “undesirable outputs” will be produced simultaneously with “desirable outputs”, this paper chooses the Super-SBM-VRS model when considering the undesirable output. Based on the combination of super efficiency Charnes–Cooper–Rhodes (CCR) model and the SBM model, this model can measure and compare the efficiency of the decision-making unit (DMU) more accurately. See Appendix A for more details of Super-SBM-VRS model.4.2.2. Semi Parametric Generalized Additive Model (SGAM).

In the existing literature, the influence of various factors on the development of industrial structure mostly uses time series and cross-sectional data, and the research methods basically follow the standard modeling steps of unit root test, co-integration test or model modification, and then panel model selection and regression. These studies basically use the method of parameter estimation, which enables calculation of the exact value of the influence coefficient, but requires a specific relationship form to be specified in advance. Typically, the relationship will be assumed to be linear, but the possibility of model misspecification from this assumption can result in errors in the conclusion. Because the influence of various factors on the development of industrial structure is more complex, there may be non-linear relationships between government investment and industrial development. This is especially likely in a rapidly-developing economy with large regional variations, such as China. In order to better explore the relationship between various factors and the development of industrial structure, this paper adopts a semi-parametric method.

The generalized additive (GAM) model proposed by Hastie and Tibshirani (1986) [31] is helpful in finding the nonlinear relationship between variables and has no limitation on the hypothesis of variable distribution, so it has strong applicability. The generalized additive model can not only capture the nonlinear relationship better, but also has the advantage that the general nonparametric model is driven by data rather than model assumptions. At the same time, it can avoid the calculation difficulty caused by the increase of dimension in the nonparametric method, commonly referred to as the dimension disaster.

In this paper, a generalized linear model is proposed:

(1) $$g\left(\eta \right)=\alpha +\beta_{1}x_{1}+\beta_{2}x_{2}+\cdots +\beta_{p}x_{p},$$

where $\eta =E\left(Y|X_{1}\cdots X_{p}\right)$ and g is the inverse of the link function. We extend this to the additive model first proposed by Stone (1985) [32], which describes the relationship between independent variable and dependent variable in nonparametric form:

(2) $$E\left(Y|X_{1}\cdots X_{p}\right)=S_{0}+S_{1}(X_{1})+S_{2}(X_{2})+\cdots +S_{p}(X_{p}),$$

where $S_{i}$, i=1,2,⋯,p are smooth functions. Combining the two methods, the generalized additive model is used to analyze the impact of government health expenditure on the development of health industrial structure. The standard form of the generalized additive model is:

(3) $$g\left(\eta \right)=S_{0}+S_{1}(X_{1})+S_{2}(X_{2})+\cdots +S_{p}(X_{p}),$$

where $\eta =E\left(Y|X_{1}\cdots X_{p}\right)$ and the inverse of g(η) are link functions. $S_{i}$, i=1,2,⋯,p are smooth functions.

It is not necessary that every term in the model is non-linear, and linear and other parametric terms can be included, so the model can be further expanded in practical application. In this way, a semi-parametric additive model can be developed by combining the forms described above:

(4) $$g(\eta )=s_{0}+\overset{q}{\underset{j=1}{\sum }}s_{j}(X_{j})+\overset{p}{\underset{j=q+1}{\sum }}\beta_{j}X_{j}+\varepsilon$$

where $\beta_{j}$ is the regression parameter of the linear part of the model.

## 5. Results

### 5.1. Efficiency Measurement of Government Health Expenditure

In this paper, the Super-SBM-VRS model is used to estimate government health expenditure efficiency.

**Figure 1:**
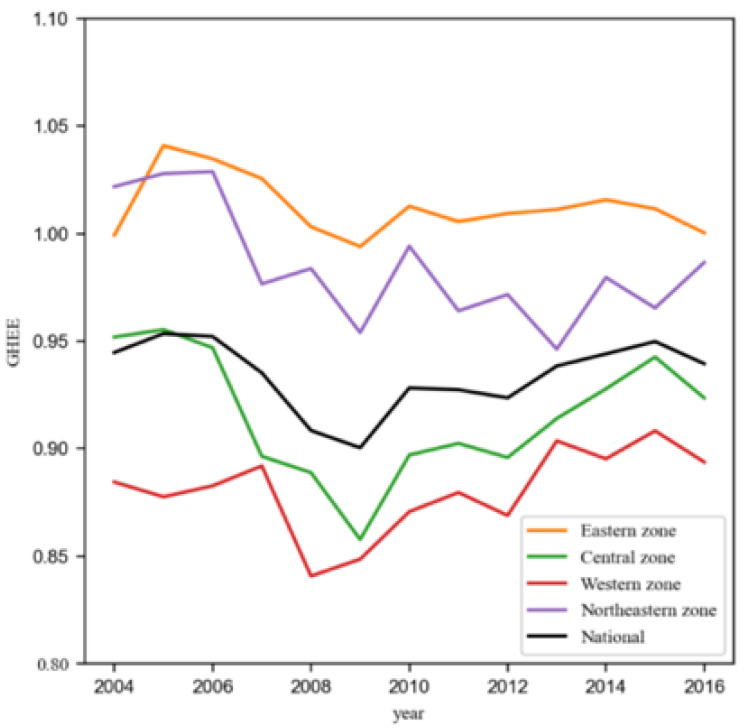
National and regional efficiency of Chinese government health expenditure in 2004–2016.

Figure 1 shows the trend in the national and regional average efficiency of government health expenditure in 25 regions of China from 2004 to 2016. Here 25 regions of China can be classified into Eastern, Central, Western, and Northeastern zones. It can be seen from this that nationally the efficiency of government health expenditure decreased slowly from 2004 to 2008, increased rapidly from 2008 to 2009, and remained level thereafter. It shows evident differences between zones, with the Eastern zone the most efficient and the Western zone the least efficient.

### 5.2. Empirical Analysis

#### 5.2.1. Single Factor Analysis

We first analyze the variables that affect the development of health industrial structure by univariate analysis, including one factor at a time in the generalized additive model to analyze predictors separately. The results show that the estimated degrees of freedom of human capital (HC), per capita private health expenditure (PPHE) and old-age dependency ratio (ODR) are 1, and the degrees of freedom of other independent variables are greater than 1. When the degree of freedom of the explanatory variable is 1, there is a linear relationship between the explanatory variable and the dependent variable. If the degrees of freedom are greater than 1, there is a nonlinear relationship between the outcome variable and the explanatory variable, and the larger the degree of freedom, the more significant the nonlinear relationship. By observing the goodness of fit and significance test of each single factor model, we found that the non-linear effect of human capital is not significant, so this paper will not analyze the non-linear effect of human capital.

#### 5.2.2. Normal Inspection and Co-Curvature Linearity Test

**Figure 2:**
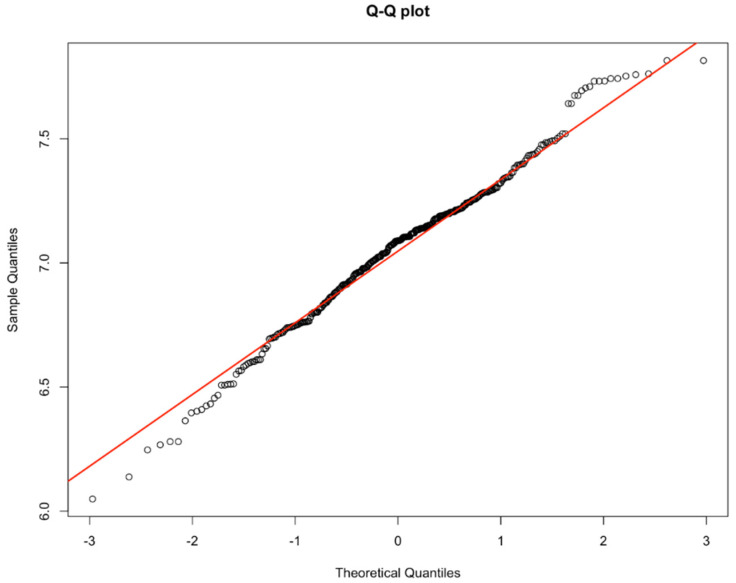
Q-Q plot of major health industrial structure development index (DI).

Before regression analysis, it is necessary to confirm whether the dependent variable is normally distributed, so as to determine whether it is suitable for the semi parametric additive model. The results of a test for normality of the industrial structure development index are shown as a Q-Q plot in Figure 2.

According to the test results, the development index of industrial structure in this paper does not obey the normal distribution, which is a positive skew distribution, so it can be calculated by the semi parametric additive model. When using the semi parametric generalized additive model, we should consider the possibility of collinearity. The results show that the correlation coefficient between the fitted values of each variable is less than 0.5. Therefore, the collinearity among the variables in this paper can be ignored.

#### 5.2.3. Semi Parametric Additive Model

In this paper, the variables are included in the model in two forms: linear and non-linear. The linear variables include per capita government health expenditure, government health expenditure efficiency, technological progress, human capital, population skewedness, per capita private health expenditure, old-age dependency ratio, economic gap, and year. It can be seen from Table 2 that both the per capita government health expenditure and the efficiency of government health expenditure have a positive impact on the development of the health industry and pass the significance test.

The results show that increases in government health expenditure per capita and in the efficiency of government health expenditure will promote the development of the health industrial structure. For every 100 yuan per person increase in local per capita government health expenditure, the industry development index will increase by 0.082 units; for every 1-unit increase in local government health expenditure efficiency, the industry development index will increase by 0.135 units. At the same time, under the influence of the scale of per capita government health expenditure and the efficiency of government health expenditure, technological progress, population bias, per capita private health expenditure, old-age dependency ratio and economic gap all have a statistically significant positive association with the development of health industrial structure. Human capital accumulation is significantly positive in the model of per capita government health expenditure, which indicates that it will have a positive impact on industrial development, but it fails to pass the statistical significance test in the model of government health expenditure efficiency, indicating that after adjusting for the efficiency of government health expenditure, human capital is not associated with industrial development.

Because the non-linear part of the semi parametric additive model is not able to fully reflect the impact of each variable on the development of the health industry, this paper will build a comprehensive semi parametric additive model to study the impact of per capita government health expenditure and the efficiency of government health expenditure on this development.

#### 5.2.4. Semi Parameter Additive Comprehensive Model

From Table 3, it can be seen that other indicators except for year have passed the significance test, under the effect of per capita government expenditure. In the model of government expenditure efficiency, the other indicators, except for human capital, have passed the significance test.

#### 5.2.5. Government Health Expenditure Scale Effect Analysis

**Figure 3:**
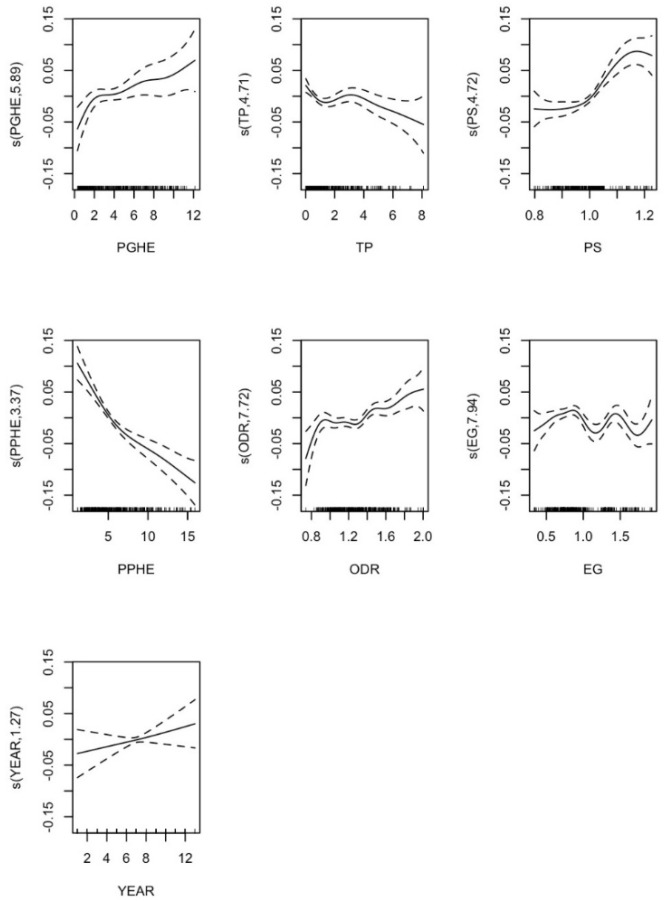
Influence of various factors and per capita government health expenditure on the development of health industrial structure.

Figure 3 shows the impact of each term in the semi-parametric model of the effect of per capita government health expenditure on industrial development. From the per capita government health expenditure (PGHE) panel of Figure 3, we can see that the non-linear impact of per capita government health expenditure on the development of health industry structure is “urgent first, then slow”. This shows that per capita government health expenditure has a positive impact on the development of the health industry. In the initial stage of spending, when government expenditure is still low, as per capita government health expenditure grows the positive impact on the development of health industrial structure is enhanced; when the per capita government health expenditure reaches about 200 yuan per person, the growth of its positive impact is gradually enhanced. When the per capita government health expenditure is between 200 yuan and 420 yuan, its positive impact on the development of the health industry is in a gentle rising stage; when the per capita government health expenditure is more than 420 yuan, this positive impact is seen to be gradually increasing, and the development of the health industry is continuously promoted.

From the technological progress (TP) subfigure, it can be seen that the non-linear impact of technological progress on the development of health industry structure shows a slow downward trend, and its impact on the development of health industry is negative. This indicates that the development of the health industry in China is still in its infancy, and the level of technological progress is far from meeting the development requirements of the structure of the health industry. From the PS-subfigure, we can see that the non-linear effect of the ratio of the permanent population to the population of registered households on the development of health industrial structure is gentle in the early stage and an inverted U curve in the later stage, indicating that when the population skewedness is lower than 1.03 the positive effect on the development of health industrial structure does not change significantly, while when the value of the ratio is between 1.03 and1.15 the promoting effect on the development of health industrial structure increases, before declining again for values greater than 1.15.

From the per capita private health expenditure (PPHE) subfigure, the non-linear effect of per capita private health expenditure on the development of health industrial structure is “first urgent and then slows down”. This shows that the development of China’s health industry is still in the primary stage, which mainly depends on stimulation from government health expenditure.

From the old-age dependency ratio (ODR) subfigure, the non-linear effect of the old-age dependency ratio on the development of the health industrial structure is of the “N” type, increasing first, then decreasing and then increasing. When the dependency ratio reaches about 0.92, the role of promoting the development of the health industrial structure gradually increases; when the dependency ratio is between 0.92 and 1.3, the impact on the development of the health industrial structure slowly drops; when the dependency ratio exceeds 1.3, the impact on the development of health industrial structure gradually increases.

From the economic gap (EG) subfigure, the non-linear effect of the economic gap on the development of health industrial structure is of the “M” type in the early stage and “U” type in the later stage. When the economic gap is about 0.55 and 1.45, the positive impact on the development of health industrial structure reaches the peak; when the economic gap is about 1.2 and 1.75, the positive impact on the development of health industrial structure is weak; when the economic gap is over 1.75, the positive impact on the development of health industrial structure has picked up.

From the year (YEAR) subfigure, we can also observe that over time the health industry continues to develop.

#### 5.2.6. Government Health Expenditure Efficiency Effect Analysis

**Figure 4:**
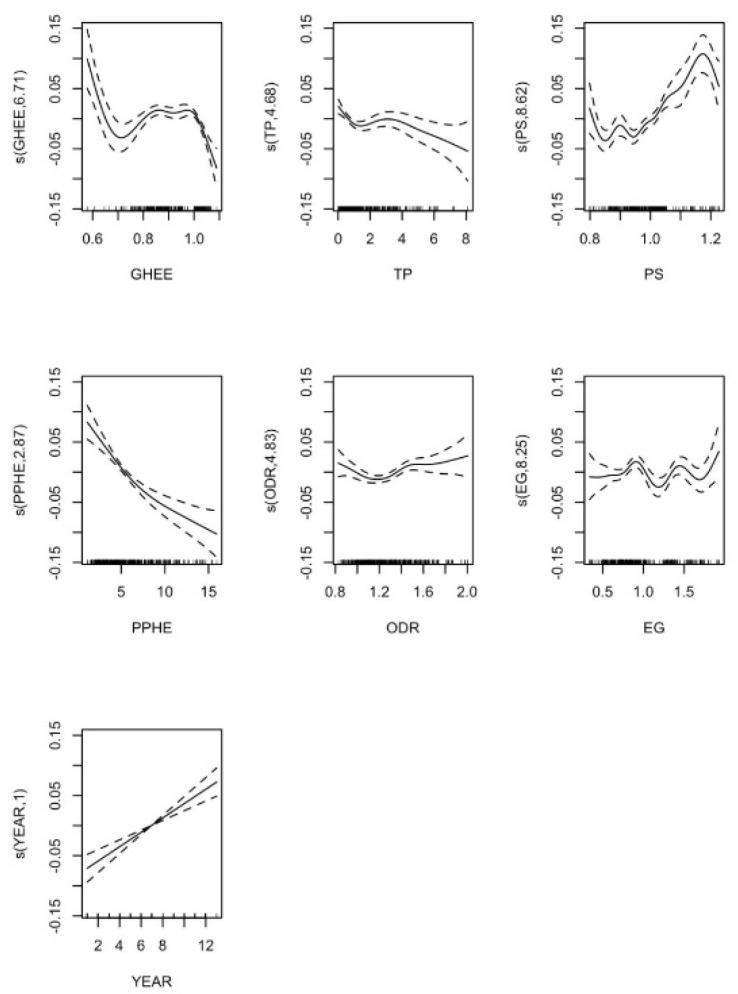
Nonlinear influence of various factors based on the efficiency of government health expenditure on the upgrading of health industrial structure.

Figure 4 shows the effect of health expenditure efficiency and other predictors in the non-linear model. When government health expenditure efficiency (GHEE) is less than 0.7, the effect on the development of health industrial structure is negative. When the efficiency of government health expenditure is between 0.7 and 1.0, the impact on the development of health industrial structure is positive, and the development of health industrial structure will be promoted rapidly with the increase of government health expenditure efficiency; when the efficiency of government health expenditure is greater than 1 and less than 1.1, the development degree of health industrial structure will decrease with the increase of government health expenditure efficiency. The influence of other factors on this development is similar to that of government health expenditure.

#### 5.2.7. Interaction

**Figure 5:**
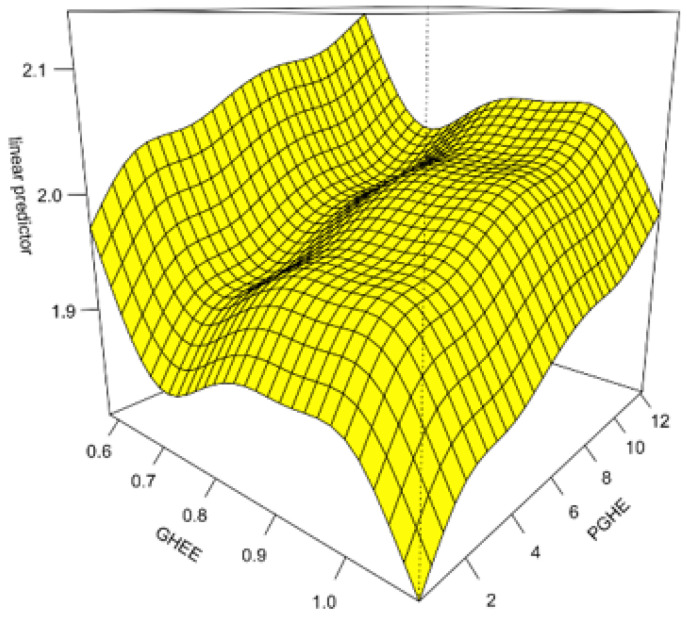
The interaction effect of per capita government health expenditure and government health expenditure efficiency on the development of health industrial structure.

In view of the common influence of the scale of government health expenditure and the efficiency of government health expenditure on the development of health industrial structure, the SGAM model can be constructed through the interaction of the two variables, so as to have a more comprehensive and in-depth understanding of the impact of government health expenditure on the development of health industrial structure. Results can be seen in Figure 5, which shows the changes in industrial structure as both efficiency and expenditure increase. With the increase in the scale of per capita government health expenditure (PGHE), the impact on the development of health industrial structure is gradually enhanced. When the efficiency of government health expenditure (GHEE) is low, its promotion of the development of health industrial structure is more obvious. It can be seen that high expenditure and low efficiency have a strong impact on the development of health industrial structure.

#### 5.2.8. Fitting Effect Test

After regression analysis, we tested the goodness of fit of this model for the validity of model estimation. In this paper, the traditional linear model and the semi parametric additive model are compared and estimated. (Appendix A)

## 6. Discussion

In recent years, China’s economy has entered a new stage of development, changing from high-speed growth to high-quality development, from extensive scale and rapid growth to quality efficiency intensive growth, and from investment-driven to innovation-driven expansion. In order to maintain sustainable growth, China must vigorously develop emerging industries and change the mode of economic development. It is essential during this growth phase to study the macroeconomic development of the health industry as a key influencer of China’s economic transformation. Based on data from 25 regions in China for the period 2004 to 2016, this paper uses a semi parametric generalized additive model to analyze a novel index measuring the macro-economic development of the health industry. It examines the effect of the scale of per capita local government health expenditure, local government health expenditure efficiency, technological progress, human capital, population bias, per capita health expenditure, aging and time on the sectoral composition of the health industry. The development of industrial structure is not only influenced by macro market policies, but also follows market rules. Its change should be multi-stage and variable in direction, such that a traditional linear model of factors affecting the development of industrial structure can only offer an incomplete perspective on the process. Therefore, based on a comprehensive perspective, this paper uses a non-linear method to measure the impact of the scale and efficiency of local government health expenditure on the macroeconomic development of the health industry.

The linear component of the model shows that the scale of per capita local government health expenditure and the efficiency of local government health expenditure have a positive impact on the development of the industry. Based on the comprehensive non-linear perspective described in this paper, the impact of per capita local government health expenditure on the development of the health industrial structure shows a positive trend of “first urgent, then slow”. The effect of government health expenditure efficiency on the development of health industrial structure can be divided into three stages.

We found that technology level did not play a significant role in promoting the development of health industrial structure. This could be because China’s health industry is still in the initial stage of development, which is mainly led by the government, so the impact of technological progress on industrial development has not been fully reflected. As a result, the consumption speed of residents is obviously slow, which leads to insufficient strength of demand-driven industrial development, and thus cannot promote the development of healthy industrial structure without government support. This finding suggests that China needs to develop better policy tools for integrating the benefits of increased investment in scientific and technological inventions into the health industry, so as to promote the optimization and development of the health industrial structure.

In this paper, the data of six provinces and cities are abnormal, which can lead to the deviation of results. Thus, to avoid unreasonable findings, we chose the data from another 25 provinces and cities.

The findings of our models suggest several policy conclusions. First, we found a strong link between government expenditure and industrial development. It is not only necessary to continue to increase the scale of local government’s health expenditure, but also to strengthen the coordination between the scale and efficiency of health expenditure, and better integrate technological developments into the healthcare industry during development, to ensure government expenditure is allocated efficiently. While early development in the industry simply required large public investment, now it is necessary to shift development policies so as to pay attention to both the scale and efficiency of expenditure and to build a development-oriented government health spending plan.

Second, we found that human capital development plays a crucial role in industrial development. In view of the characteristics of the current low stock and low quality of human capital, the government should increase investment in human capital; continue to increase investment in education, especially in higher education and vocational education, and train high-quality and high-tech personnel so as to strengthen the flow of talent from the primary and secondary sectors to the tertiary sector; promote the optimization of regional human capital structure and the development of health industry; and provide a highly-skilled workforce in support of the development of the industrial structure.

Third, our model found that skewedness in population aging had an important effect on industrial development. Given this, steps are now needed to encourage development of the old-age care industry and promote the development of this component of the health sector. In recent years, the proportion of the aged population in China has risen sharply, which has given birth to the “old age economy” [33]. In order to seize the economic opportunity brought by aging, we should follow the trend of population structure change, vigorously develop a health industry suitable for the old-age, and improve the consumption impact of the old-age population.

Finally, we found strong effects of migration patterns on industrial development, suggesting the need for a sustainable development mechanism. China should speed up the reform of the household registration system, and accelerate the process of urbanization. The gathering and flow of population has led to the development of industries and the emergence of high-tech sectors within health that will absorb human resources, while the traditional labor-intensive industries will be gradually eliminated.

## 7. Conclusions

In this paper, we found that the scale and efficiency of government health expenditure have a dynamic impact on the development of health industrial structure. The scale of local government health expenditure is conducive to promoting the process of development of the health industrial structure. However, our model also finds that balanced efficiency in expenditure allocation is also important. China’s health industry is still developing, but is essential for both continued economic growth and to enhance the welfare of the population. By paying careful attention to the relationship between government expenditure, efficiency of expenditure, and key demographic and social factors operating in the economy, it is possible to continue to develop China’s health industry efficiently and rapidly, so that it can play a full role in the economy as both a driver of growth and to strive for the goal of health for all.

## Figures and Tables

**Table 1:** Descriptive Statistics.

Variable	*N*	Max	Min	Mean	Std. Deviation
Industrial development index (DI)	325	7.815	6.280	7.076	0.291
Per capita government health expenditure (PGHE)	325	12.153	0.295	4.154	3.040
Efficiency of local government health expenditure (GHEE)	325	4.260	0.579	0.972	0.283
Technical progress (TP)	325	8.100	0.014	1.496	1.627
Human Capital (HC)	325	9.972	6.378	8.441	0.706
Population skewedness (PS)	325	1.227	0.798	0.985	0.078
Per capita private health expenditure (PPHE)	325	15.932	1.110	6.027	3.290
Old-age Dependency Ratio (ODR)	325	2.004	0.740	1.272	0.243
Economic gap (EG)	325	1.928	0.337	0.957	0.360
Year (YEAR)	325	13	1	7	3.741

**Table 2:** Impact of each predictor on the development of health industrial structure under the semi parametric additive model.

**Variable**		**Estimate**	**Std. Error**	***p*-Value**	**Estimate**	**Std. Error**	***p*-Value**
	**Linear Part**						
(Intercept)		0.14	0.005	<0.001	0.16	0.004	<0.001
PGHE		0.08	0.009	<0.001	\		
GHEE		\			0.13	0.004	<0.001
TP		0.04	0.009	<0.001	0.05	0.008	<0.001
HC		0.01	0.006	0.015	0.00	0.006	0.188
PS		0.13	0.005	<0.001	0.16	0.005	<0.001
PPHE		0.05	0.005	<0.001	0.07	0.004	<0.001
ODR		0.18	0.009	<0.001	0.20	0.006	<0.001
EG		0.14	0.012	<0.001	0.15	0.012	<0.001
YEAR		0.07	0.004	<0.001	0.08	0.003	<0.001
**Variable**		**edf**	**Ref.df**	***p*-Value**	**edf**	**Ref.df**	***p*-Value**
	**Nonlinear Part**						
s(PGHE)		5.10	6.28	<0.001	\		
s(GHEE)		\			6.70	7.81	<0.001
s(TP)		4.02	5.09	<0.001	4.00	5.08	<0.001
s(PS)		4.71	5.80	<0.001	8.62	8.94	<0.001
s(PPHE)		2.67	3.57	<0.001	2.19	2.97	<0.001
s(ODR)		7.67	8.53	<0.001	4.78	5.89	<0.001
s(EG)		7.83	8.56	<0.001	8.14	8.72	<0.001
s(YEAR)		0.58	0.79	<0.001	0.31	0.31	<0.001

**Table 3:** Hypothesis test results of comprehensive effects of predictors on the development of health industrial structure.

**Variable**		**Estimate**	**Std. Error**	***p*-Value**	**Estimate**	**Std. Error**	***p*-Value**
	**Linear Part**						
HC		0.015	0.006	0.015	0.008	0.006	0.188
**Variable**		**edf**	**Ref.df**	***p*-Value**	**edf**	**Ref.df**	***p*-Value**
	**Nonlinear Part**						
s(PGHE)		5.89	7.07	0.031	\		
s(GHEE)		\			6.71	7.82	<0.001
s(TP)		4.72	5.80	<0.001	4.68	5.76	<0.001
s(PS)		4.71	5.78	<0.001	8.62	8.95	<0.001
s(PPHE)		3.37	4.27	<0.001	2.87	3.66	<0.001
s(ODR)		7.72	8.58	<0.001	4.83	5.94	0.001
s(EG)		7.94	8.67	<0.001	8.25	8.83	0.006
s(YEAR)		1.27	1.48	0.317	1	1	<0.001

**Figure A1:**
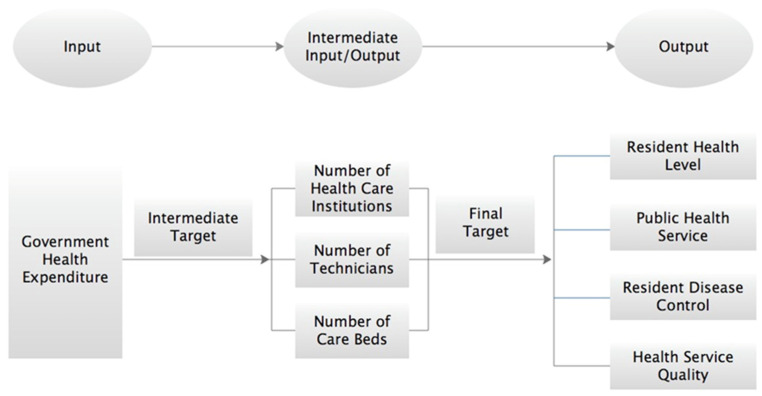
Government Health Expenditure Target.

## Appendix A

### Appendix A.1. Dependent Variable

We use a method for measuring structural change put forward by John H. Moore [23]. Moore’s index uses the method of space vector measurement, based on an angle in vector space, and divides the industry into n sectors. A group of n-dimensional vectors is formed, and the angle between the two groups of vectors in two periods is taken as the index of the degree of industrial structure change, which is called Moore’s structure change value.

The calculation formula is as follows:

(A1) $$M_{i}^{+}=\sum_{i=1}^{n}W_{i,t}\cdot W_{i,t+1}/{\left(\sum_{i=1}^{n}W_{i,t}^{2}\right)}^{1/2}\cdot {\left(\sum_{i=1}^{n}W_{i,t+1}^{2}\right)}^{1/2},$$

(A2) $$\cos\theta =M_{i}^{+},$$

(A3) $$\theta =\mathrm{arc}\cos M_{i}^{+},$$

Among them, $M_{i}^{+}$ is the change value of Moore structure; $W_{i,t}$ is the proportion of the economy taken up by the ith industry in phase t, $W_{i,t+1}$ is the proportion of the ith industry in the period t + 1, and θ is the degree of change of Moore’s structure. This paper studies the industrial structure of three industries, so in this paper *n* = 3.

Moore’s structural change value measures the degree of industrial structure change between the two periods. The smaller the value of $M_{i}^{+}$ or the greater the value of θ, the greater the change degree of industrial structure. Both of them can directly reflect the change of industrial structure, but can not reflect the direction and trend of industrial structure evolution. In order to measure the change of industrial structure from low level to high level, this paper further constructs an industrial development index.

The index used in this paper measures the process of changing the focus of health industrial structure from the primary industry to the secondary and tertiary sectors with the development of the economy. To do this we establish a three-dimensional coordinate system, with basis vectors $X_{1}={\left(1,0,0\right)}^{T}$, $X_{2}={\left(0,1,0\right)}^{T}$ and $X_{3}={\left(0,0,1\right)}^{T}$ which represent a health industry in which the three sectors are in an extreme state. In this condition the vector $X_{i}\;\left(i=1,2,3\right)$ indicates that there is only the ith industry in the region. The actual health industrial structure in a region is represented numerically by a vector $X_{0}={\left(x_{1,0},x_{2,0},x_{3,0}\right)}^{T}$, where $x_{i,0}$(0$\leq x_{i,0}\leq 1,i=1,2,3)$ is the proportion of the total output taken by the i-th industry in the region, satisfying $x_{1,0}+x_{2,0}+x_{3,0}=1$. We define the angle between $X_{0}$ and $X_{i}$ as $\theta_{i}\;\left(i=1,2,3\right)$, so that its value represents the industrial structure of the region, where $\theta_{i}$ is determined by the following formula:

(A4) $$\theta_{i}=\mathrm{arc}\cos\left(\frac{X_{0}\cdot X_{i}}{\left|X_{0}\right|\left|X_{i}\right|}\right),$$

Therefore, the development index DI of health industrial structure development is the cumulative sum of these angles:

(A5) $$\mathrm{DI}=3\theta_{1}+2\theta_{2}+\theta_{3},$$

It can be noted that the larger the DI, the higher the level of industrial structure development.

### Appendix A.2. Efficiency of Government Health Expenditure Variable Selection

#### Appendix A.2.1. Input Variable Selection

In view of the lack of data in Beijing, Tianjin, Shanghai, Hainan, Qinghai and Tibet, this paper selects 25 other regions in China from 2004 to 2016 as data samples (see Table A1).

**Table A1:** Chinese local government health expenditures from 2004 to 2016.

	Time	2004	2005	2006	2007	2008	2009	2010	2011	2012	2013	2014	2015	2016
Variable														
**Local GHE** (1000 million Chinese yuan)		69.7	85.6	109.6	166.6	234.1	349.2	420.8	571.9	645.5	740.4	912.1	107.4	1179.6

#### Appendix A.2.2. Dependent and Explanatory Variables

The basic goal of government health expenditure (GHE) is to achieve the optimal supply of public health services under the condition of limited health resources, to obtain the maximum health output. From this point of view, we can divide GHE into intermediate and final targets (see Figure A1) [24].

Health expenditure is measured using the super efficiency non radial model (SBM-VRS Model) to measure government health expenditure efficiency (GHEE). Therefore, according to the final goal of local GHE, this paper chooses the following seven indicators to show the input and output of local governments in the field of health services: (1) severe malnutrition rate among children under 5 years old and mortality rate, reflecting the health level of residents; (2) the number of outpatient visits and the medical expenses per capita of outpatients and inpatients, reflecting public health services; (3) infectious disease incidence rate of Class A and B statutory reports and infectious disease mortality rate, reflecting the residents’ diseases control; (4) hospital bed utilization rate, reflecting the quality of health care services. The output of local government in public health service from 2004 to 2016 is shown in Table A2.

**Table A2:** Outputs of government-invested public health service from 2004 to 2016.

Indicator Name	Maximum	Minimum	Mean	Standard Deviation
Outpatient consultation (100 million)	8.12	0.07	1.75	1.71
Infectious disease incidence rate of Class A and B statutory reports (%)	738.19	102.48	266.65	97.60
Outpatient and inpatient medical expenses per capita (Chinese yuan)	11,591.5	1091.5	5932.38	1908.34
Bed use rate (%)	99.3	54.7	81.68	10.03
Severe malnutrition rate among children under 5 years old (%)	5.57	0.26	1.68	1.08
Population mortality rate (%)	7.28	4.21	6.04	0.711
Infectious disease mortality rate (%)	7.35	0.07	1.42	1.75

### Appendix A.3. Super Efficiency Slacked Based Model (SBM) under the Variable Returns to Scale (Super-SBM-VRS) Model

The Data Envelopment Analysis (DEA) method, occasionally called frontier analysis, was developed by Charnes, Cooper and Rhodes (CCR) in 1978 [29]. DEA models are classified into Banker, Charnes, and Cooper (BCC) [34] model. The CCR model is the earliest DEA model and also the most basic. The DEA effective decision-making unit obtained by the CCR model is technology efficient and scale effective at the same time. The CCR model can not only evaluate the technical efficiency of Decision-Making Units (DMUs). Therefore, a BCC model is proposed to evaluate the technical effectiveness of DMUs. The BCC model is mostly used to study the efficiency of “diseconomies of scale” sectors, and can only be used to explain the change of technical efficiency. Both models do not consider input and output dynamically from the relaxation measure of variables, resulting in large or small deviation between the theoretical value and the actual value of the measurement.

The Slack Based Measure (SBM) takes into account the slack variables in input and output, and the final value of efficiency measured by the SBM model is a strict decreasing function of input and output slack variables.

The SBM model is constructed as:

(A6) $$min\rho =\frac{1-\frac{1}{m}\sum_{i=1}^{m}\frac{s_{i}^{-}}{x_{ik}}}{1+\frac{1}{q}\sum_{r=1}^{q}s_{r}^{+}/y_{rk}},$$

(A7) $$s.t.\{\begin{array}{c} x_{k}=X\lambda +s^{-}\\ y_{rk}=Y\lambda -s^{+}\\ s^{-}\geq 0,s^{+}\geq 0,\lambda \geq 0 \end{array},$$

In the SBM model, minρ is used to express the efficiency value of DMU, $s_{i}^{-}$ is used to express the redundancy of input in i, $s_{r}^{+}$ is used to express the deficiency of output in r, $x_{ik}$ is the i input of the kth DMU, $y_{rk}$ is the r output of the kth DMU, λ is the adjustment matrix, Xλ is used to express the input on the frontier, and Yλ is used to express the output on the frontier. In the unguided model, input and output data cannot be 0.

The objective function minρ is strictly decreasing with respect to $s^{-}$, $s^{+}$ and 0≤minρ ≤1. Because of this, for a specific evaluated unit, it is efficient when and only when minρ=1, i.e., $s^{-}=0,s^{+}=0$. If minρ<1, the evaluated unit is inefficient. There is a need for improvement.

Among them, input inefficiency (II) (i.e., input redundancy rate) and output inefficiency (OI) are as follows:

(A8) $$II=\frac{1}{m}\sum_{i=1}^{m}\frac{s_{i}^{-}}{x_{ik}},$$

(A9) $$OI=\frac{1}{q}\sum_{r=1}^{q}s_{r}^{+}/y_{rk},$$

When the final efficiency value of multiple DMUs is completely effective, that is, the final value is 1, the efficiency of these DMUs cannot be further measured and evaluated. In order to make up for the above defects, a super efficiency SBM model is proposed, that is, when there are more than one DMU, the DMU is in effective state, the efficiency value between these DMUs can be further identified. After deleting the evaluated effective DMUs from the production possibility set (PSS), measuring the distance between each DMU and PSS is called super efficiency. These efficiency values may be equal to or greater than 1, which is conducive to efficiency comparison among different DMUs.

The Super-SBM model is as follows:

(A10) $$min\rho_{SE}=\frac{1+\frac{1}{m}\sum_{i=1}^{m}s_{i}^{-}/x_{ik}}{1-\frac{1}{s}\sum_{r=1}^{s}s_{i}^{+}/y_{rk}},$$

(A11) $$s.t.\{\begin{array}{c} \overset{n}{\underset{j=1,j\neq k}{\sum }}x_{ij}\lambda_{j}-s_{i}^{-}\leq x_{ik}\\ \;\overset{n}{\underset{j=1,j\neq k}{\sum }}y_{rj}\lambda_{j}+s_{i}^{+}\leq y_{rk}\\ \lambda ,s^{-},s^{+}\geq 0\\ i=1,2,\cdots ,m;\;r=1,2,\cdots ,q;\;j=1,2,\cdots ,n\left(j\neq k\right) \end{array},$$

In practice, considering that some “undesirable outputs” will be produced simultaneously with “desirable outputs”, this paper chooses the super efficiency SBM-VRS model considering the undesirable outputs. Based on the combination of super efficiency CCR model and SBM model, this model can measure and compare the efficiency of DMU more accurately.

Super-SBM-VRS model is to add constraints $\overset{n}{\underset{j=1,j\neq k}{\sum }}\lambda_{j}=1$ on this basis.

### Appendix A.4. Model Diagnostics

After regression analysis, we tested the goodness of fit of this model to test the validity of model estimation. In this paper, the traditional linear model and the semi parametric additive model are compared and estimated. The Akaike information criterion (AIC) value and the sum of squared residuals of the estimated results of the two models are shown in Table A3. It can be seen from the table that the AIC value and residual adjustment and results of the semi parametric generalized additive model are significantly smaller than those of the traditional linear model, showing that the fitting effect of the semi parametric additive model is more accurate than that of the traditional linear model.

**Table A3:** Comparison of AIC test and residual sum of squares between traditional linear model and semi parametric additive model.

Variables	Traditional Linear Model		SGAM	
	Explanatory Variable: Scale of Local Government Health Expenditure	Explanatory Variable: Local Government Health Expenditure Efficiency	Explanatory Variable: Scale of Local Government Health Expenditure	Explanatory Variable: Local Government Health Expenditure Efficiency
AIC	−1188.106	−1171.849	−1270.926	−1314.654
Sum of squares of residuals	0.462	0.432	0.301	0.228
